# Systematic identification of Celastrol-binding proteins reveals that Shoc2 is inhibited by Celastrol

**DOI:** 10.1042/BSR20181233

**Published:** 2018-11-21

**Authors:** Huang Xiao-pei, Chen Ji-kuai, Wei Xue, Yi-Fan Dong, Lang Yan, Zhang Xiao-fang, Pan Ya-min, Chang Wen-jun, Zhu Jiang-bo

**Affiliations:** 1 Department of Health Toxicology, Faculty of Naval Medicine, Second Military Medical University, Shanghai 200433, P.R. China; 2 Department of Environmental Hygiene, Faculty of Naval Medicine, Second Military Medical University, Shanghai 200433, P.R. China; 3 The First Department of Endoscopy, Shuguang Hospital Affiliated to Shanghai University of Traditional Chinese Medicine, Shanghai 201203, P.R. China

**Keywords:** Celastrol, Colorectal cancer, ERK, Human proteome microarray, Shoc2

## Abstract

Colorectal cancer (CRC) is the third most commonly diagnosed cancer. Celastrol exhibits anti-tumor activities in a variety of cancers. However, the effect of Celastrol on human CRC and the underlying mechanisms still need to be elucidated. The present study aimed to use *in vitro* and *in vivo* methods to clarify the anti-tumor effect of Celastrol and use protein microarrays to explore its mechanisms. We demonstrated that Celastrol effectively inhibited SW480 CRC cell proliferation. Two weeks of Celastrol gavage significantly inhibited the growth of xenografts in nude mice. A total of 69 candidate proteins were identified in the protein microarray experiment, including the most highly enriched protein Shoc2, which is a scaffold protein that modulates cell motility and metastasis through the ERK pathway. Celastrol significantly inhibited ERK1/2 phosphorylation in cell lines and xenograft tumors. Down-regulation of Shoc2 expression using Shoc2 siRNA also inhibited ERK1/2 phosphorylation. Furthermore, down-regulation of Shoc2 expression also significantly inhibited proliferation, colony formation, and migration functions of tumor cells. In addition, the LD0 of Celastrol by gavage is equal or more than 80 mg/kg in C57 male mice. In summary, we unraveled the anti-CRC function of Celastrol and confirmed for the first time that it inhibited the ERK1/2 pathway through binding to Shoc2.

## Introduction

Colorectal cancer (CRC) has the third highest incidence rate among cancers worldwide and is the second cause of cancer death [1,2]. Currently, surgical resection combined with chemotherapy is a therapeutic strategy for CRC patients [3,4]. However, the severe adverse reactions and dose-limiting toxicities of chemotherapeutic drugs not only reduce the quality of life of patients but also prompt them to refuse to continue chemotherapy [5,6]. Therefore, screening of anti-CRC components that are less toxic from traditional Chinese herbal medicines may be an important step to promote cancer treatment.

Celastrol is a triterpenoid drug and is a pharmacologically active component extracted from the Chinese herbal plant *Tripterygium wilfordii*. Celastrol exerts various pharmacological and physiological activities in chronic inflammation, autoimmune diseases, and neurodegenerative diseases [7–9]. Studies indicated that Celastrol exhibits anti-tumor effects in different types of cancers (including prostate cancer, glioma, osteosarcoma, and liver cancer) [10–15]. Celastrol is a cytotoxic agent and studies on its anti-tumor mechanisms mainly focus on apoptosis induction [16–19]. However, these studies only compared cell outcomes after Celastrol treatment but did not confirm Celastrol-binding proteins and the specific mechanisms of action.

In the present study, we applied biotin-labeled Celastrol and a human proteome microarray system containing 16,368 proteins to identify Celastrol-binding proteins. We confirmed 69 candidate proteins. Surprisingly, we found that Shoc2, which is associated with tumorigenesis, formed the strongest bond with Celastrol. Celastrol inhibited phosphorylation of the Shoc2 downstream protein ERK. Down-regulation of Shoc2 expression also inhibited tumor cell proliferation and migration. Therefore, our study suggested that Shoc2 was a functional target of Celastrol in cancers. Our results provided new explanations for the inhibitory function of Celastrol in other cancers.

## Materials and methods

### Probe of Biotin-Celastrol on the human proteome microarray

Biotin-Celastrol was synthesized and purified in Shanghai Simr Biotech Co., Ltd. (Simr, Shanghai, China) (Supplementary Material S1). Proteome microarray assay was carried out as previously described. Briefly, Biotin-Celastrol was diluted to 10 μM in blocking buffer and incubated on the proteome microarray at room temperature for 1 h. The microarrays were washed with PBST three times for 5 min each and were incubated with Cy5-streptavidin at 1:1000 dilution (Sigma) for 1 h at room temperature, followed by three 5-min washes in PBST. The microarrays were spun dry at 250×***g*** for 3 min and were scanned with a GenePix 4000B microarray scanner (Axon Instruments) to visualize and record the results. GenePix Pro-6.0 software was used for data analysis.

### Cell culture and treatment

Human colorectal cancer cell line SW480, HT-29, and LoVo were purchased from the American Type Culture Collection. The cell line was maintained in DMEM (SW480 and HT-29, Gibco, BRL) or F-12K Medium (LoVo) supplemented with 10% FBS (GIBCO) and 1% streptomycin/penicillin (GIBCO) in a humidified atmosphere of 95% air plus 5% CO_2_ at 37°C.

### siRNA transfection

Cells were seeded in six-well plates at a concentration of 1.5 × 10^5^ and cultured in a medium without antibiotics for approximately 24 h before transfection. The siRNA sequence was synthesized by Genepharma Company (Shanghai, China) and the sequence of SHOC2 siRNA used was 5′-AUACAGAUGUACGAGUCCATT-3′ while the control was 5′-UUCUCCGAACGUGUCACGUTT-3′. With Lipofectamine RNAiMAX reagent (Invitrogen, Carlsbad, CA, U.S.A.), the siRNAs were transfected into SW480 cell with a final concentration of 20 nM following the manufacturer’s instructions. Expressions of Shoc2 in the siRNA and control-transfected cells was determined by Western blotting.

### Immunoprecipitation and streptavidin agarose affinity assay

For immunoprecipitations, cells were lysed on ice for 30 min in RIPA buffer. Cleared lysates were separated by 10% SDS–PAGE, transferred onto NC membranes and then blocked for 2 h at room temperature with 5% nonfat dried milk. Protein detection was accomplished by probing the membranes with β-actin antibody, Shoc2 antibody, p-ERK antibody, and ERK antibody (Cell Signaling Technology, Beverly, MA) and exposed with an Amersham Imager 600 (GE Healthcare Bio-Sciences AB, Uppsala, Sweden). ImageJ software was then used to scan and quantify the immunoblots.

Biotin pull-down assays were carried out by incubating 500 μg of cell lysates with 5 μg of Biotin-Celastrol for 1 h at room temperature. Complexes were isolated with streptavidin agarose column (Pierce Biotechnology, Rockford, IL), and bound proteins in the pull-down material were analyzed by Western blotting by using monoclonal antibodies. After secondary antibody incubations, signals were visualized by enhanced chemiluminescence.

### Cell proliferation assay

Cells were seeded (*n*=1500 cells per well), in triplicate in a 96-well microtiter plate in 100 μl complete medium. Cell activity was detected every 24 h up to a period of 5 day by using Cell Counting Kit-8 (Dojindo, Kumamoto, Japan). At the desired time points, 10 μl Cell Counting Kit-8 solution was added to each well. The plate was incubated at 37°C in a CO_2_ incubator for 1 h, then the optical density (OD) was measured on an enzyme-linked immunosorbent assay plate reader at wavelength of 450 nm. A growth curve was prepared from three independent experiments by plotting OD at 450 nm (on *y*-axis) against time (on *x*-axis).

### Colony-forming assay

Cells (*n*=1000) were plated in 60-mm tissue culture plates in triplicate. Cells were grown in complete DMEM medium for 14 days, with medium changed every 2–3 days. Cells were fixed with 4% paraformaldehyde for 1 h, which was followed by staining with crystal violet for 1 h at room temperature. Stained plates were then washed with running water for 1 min and dried in the air, and images were captured using a high-resolution Nikon D70 camera (Nikon, Tokyo, Japan).

### Migration assay

Cells were harvested and re-suspended in serum-free DMEM medium. For the migration assay, 2.5 × 10^4^ cells were added into the upper chamber of the insert (BD Bioscience, 8 μm pore size). Cells were plated in medium without serum, and medium containing 20% FBS in the lower chamber served as the chemoattractant. After 6 h of incubation, cells were fixed with 4% formaldehyde and stained with crystal violet staining solution, and cells on the upper side of the insert were removed with a cotton swab. The migratory capacity was evaluated as the total number of cells on the lower surface of the membrane, as determined by microscopy.

### Tumor formation in nude mice

To test the tumorigenicity of the cells, NMRI nude mice (Shanghai SLAC Laboratory Animal Co., Ltd., Shanghai, China) were used. SW480 (5 × 10^6^) cells suspended in PBS were injected subcutaneously in the dorsal flank of 5-week-old NMRI nude mice. Four mice were injected and observed for 3 weeks for tumor formation. After tumor formation, nude mice were randomly divided into two groups and were given Celastrol (3 mg/kg) and solvent (10% DMSO, 70% cremophor/alcohol [3:1], and 20% PBS) using the method of intragastric administration, respectively. After 15 consecutive days of gavage, the mice were sacrificed and the xenograft tumors were harvested and examined. Tumor formation process was observed continuously and tumor volume was measured every three days. All animal procedures used in the present study were approved by the Institutional Animal Care and Use Committee of the Second Military Medical University.

### Acute toxicity study

To assess the toxicity profile of the Celastrol, the animals were administered the Celastrol (80, 40, 20, or 0 mg/kg) and were monitored for 14 days. The solvent formulation: 10% DMSO, 70% cremophor/alcohol (3:1), and 20% PBS.

### Statistical analysis

The data are expressed as the mean ± S.E.M. and analyzed for statistical significance using GraphPad Prism 5.0.1 (GraphPad Software, La Jolla, CA, U.S.A.). One-way ANOVA was used to detect statistical significance among group means and Bonferroni *post-hoc* analysis was used to compare specific groups when ANOVA showed significant differences. *P*<0.05 was considered to be statistically significant.

## Results

### Celastrol attenuates the tumorigenicity of colon cancer cell *in vivo* and *in vitro*

**Figure 1:**
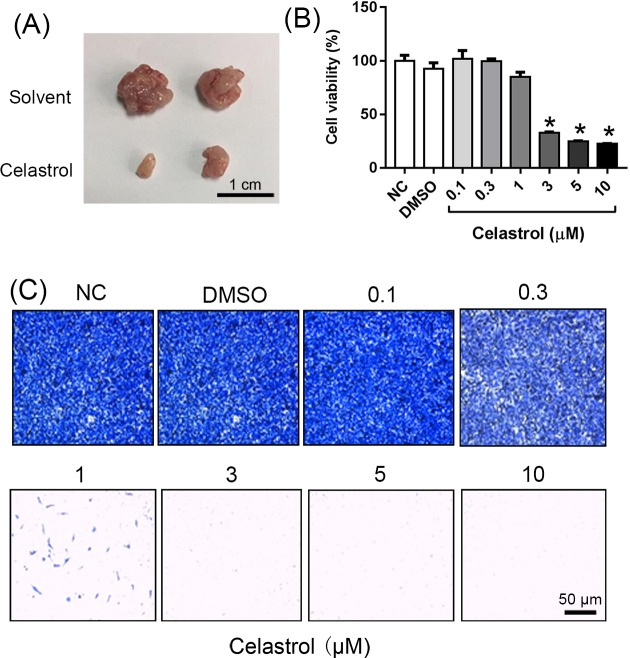
Celastrol attenuates the tumorigenicity of colon cancer cell *in vivo* and *in vitro* (**A**) Representative images of tumor tissues harvested from different groups. Mice were given Celastrol by gavage at a dose of 3 mg/kg daily for 15 days, *n*=4. The solvent formulation: 10% DMSO, 70% cremophor/alcohol (3:1), and 20% PBS. (**B**) Quantitative data of cell viability under different concentrations of Celastrol treatments for 24 h, *n*=6, *P<0.001 vs. DMSO group. (**C**) SW480 cells were transfected with different concentrations of Celastrol. Cell migration was assessed after 24 h incubation by transwell assay, *n*=6.

The anti-tumor effect of Celastrol was evaluated on BALB/c-nu/nu mice. SW480 cells were inoculated into nude mice to establish the subcutaneous tumor models. As shown in Figure 1A, there existed significant increases in tumor volumes in the groups treated with PBS. However, the group treated with Celastrol showed significantly slowed-down tumor growth in comparison with the PBS groups. As shown in Figure 1B, 0.1–10 µM Celastrol decreased cell viability in a dose-dependent manner (Figure 1B), which was significant at 3–10 µM. In addition, considering SW480 cells are highly metastatic, we wonder whether Celastrol has any effect on migration capacity. Migration chamber assay was used to verify the biological function of Celastrol in colon cancer cell migration. As the representative micrographs clearly demonstrate, Celastrol led to potent inhibition of cell migration (Figure 1C).

### Identification of Celastrol-binding proteins by protein microarray chip

**Figure 2:**
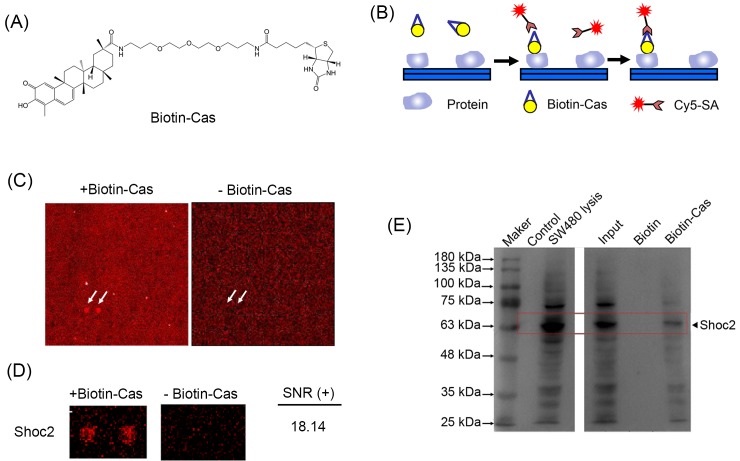
Identification of Celastrol binding proteins (**A**) Chemical structure of Biotin-Cas. (**B**) A schematic representation of identification of Biotin-Cas binding protein using a proteome microarray. (**C**) Images of one picked block from the same location of both of the experimental microarrays (left) and the biotin control (right). (**D**) Images of Biotin-Cas binding protein candidate Shoc2 in the proteome microarray. (**E**) SW480 cell lysis was treated with Biotin-Cas for 1 h and the elution, prey flow, and wash medium were subjected to Western blot using the Shoc2 antibody.

To identify Celastrol-binding proteins, a human proteome microarray consisting of 16368 affinity-purified N-terminal GST-tagged proteins was employed with a biotinylated Celastrol molecule (Figure 2A) [17]. Briefly, the celastrol-biotin conjugate (Biotin-Cas) was incubated with the human proteome microarray, and proteins with Celastrol-binding capacity were identified by adding Cy5-conjugated streptavidin (Cy5-SA) (Figure 2B). Free biotin was included to avoid false-positive detections. Chosen blocks from the same location in the experimental microarray and the negative control microarray are shown in Figure 2C. In total, we identified 69 candidate Celastrol-binding proteins from these micro arrays (Supplementary Material S2). Clearly, two positive signals are present on the microarray incubated with Biotin-Cas than on the free biotin microarray. Shoc2 were identified as potential target protein of Biotin-Cas. Representative spots of candidate protein were shown in Figure 2D. The signal-to-noise ratio (SNR) for Shoc2 was 18.14. To confirm Celastrol–Shoc2 interaction, the SW480 cell lysis was treated with Biotin-Cas for 1 h in spin columns. Binding proteins were subjected to Western blot assay by Shoc2 antibody. We found that Shoc2 was pulled down by Biotin-Cas (Figure 2E), suggesting the binding between Celastrol and Shoc2.

### Celastrol reduces phosphorylation of ERKs *in vitro* and *in vivo*

**Figure 3:**
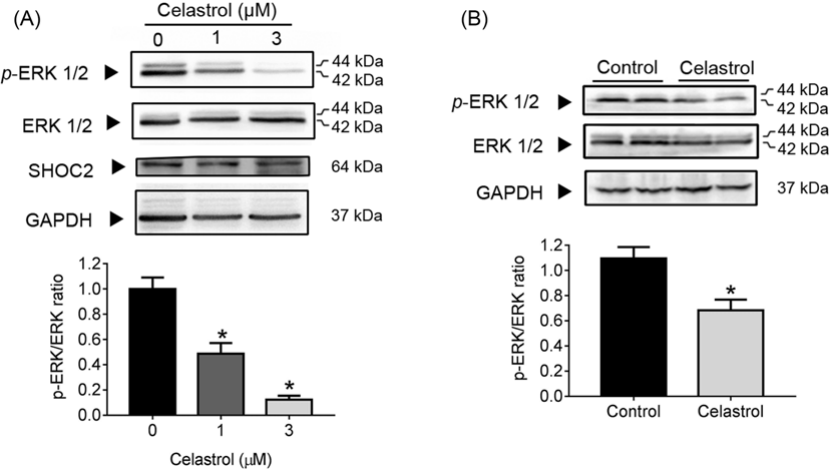
Celastrol reduces phosphorylation of ERKs *in vitro* and *in vivo* (**A**) The SW480 cells were treated with Celastrol at indicated concentrations for 24 h, lysed, and Western blot was performed using indicated antibodies. (**B**) The effect of the treatment with Celastrol on ERKs phosphorylation were measured in multiple samples (*n*=6) of xenografts by Western blot. *P<0.001 vs*.* control group.

Shoc2 was identified as a gene playing a positive role in mitogen-activated protein kinase (MAPK)/ERK pathway activation [18]. Up-regulation of Shoc2 during human melanoma metastasis also resulted in the ERK phosphorylation [20]. We examined the influence of Celastrol on the phosphorylation of ERKs by Western blot. Intriguingly, ERK1/2 phosphorylation showed a dose-dependent decrease in Celastrol-treated SW480 cells compared with control cells (Figure 3A). Moreover, the ERK1/2 phosphorylation in transplanted tumors in the Celastrol groups was lower than those of control group (Figure 3B). These results validated Celastrol as an inhibitor of the phosphorylation of the PERK1/2 in SW480 cell-transplanted tumors, which might be responsible for the inhibition of the xenograft growth.

### Shoc2 knockdown reduces phosphorylation of ERKs in SW480 cells

**Figure 4:**
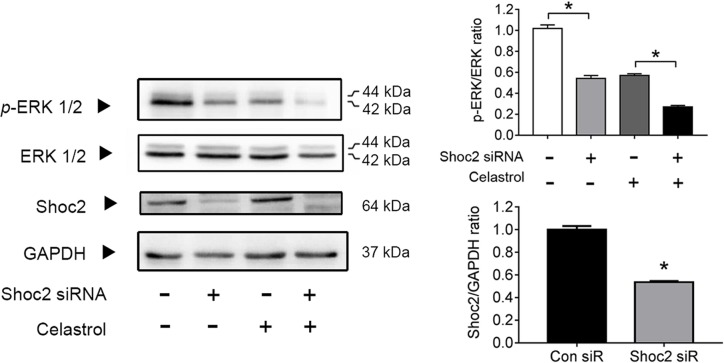
Shoc2 knockdown and Celastrol reduces phosphorylation of ERKs in SW480 cells SW480 cells were infected with Shoc2 siRNA for 72 h and then with Celastrol 1 μM for 24 h. The WCLs of the control cells and cells with Shoc2 knockdown were immunoblotted against the indicated proteins. *P<0.001 vs. control group.

Because we observed the reduction of ERKs phosphorylation by Celastrol, we investigated whether Shoc2 knockdown can reduce phosphorylation of ERKs. Immunoblotting analyses showed that expression of Shoc2 was significantly reduced by Shoc2 siRNA in SW480 cells (Figure 4). Furthermore, Celastrol and the knockdown of Shoc2 in SW480 cells significantly down-regulated phosphorylation of ERK1/2 (Figure 4).

### Shoc2 knockdown leads to reduction in cell proliferation and migration

**Figure 5:**
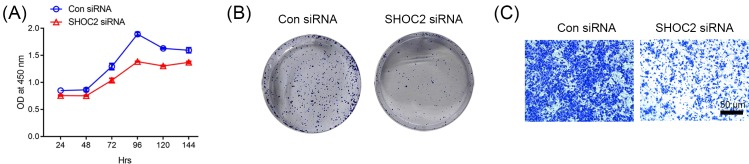
Shoc2 knockdown leads to reduction in cell proliferation and mobility (**A**) Effect of Shoc2 knockdown on cell growth. Cell proliferation curves of Shoc2 siRNA and control siRNA using CCK-8 assay. Cell proliferation was plotted against time. Results are mean ± S.E.M. of three independent experiments performed in triplicate. (**B**) Note decreased cell proliferation in Shoc2 knockdown clones. Colony-forming assay in Shoc2 knockdown cells. Representative images of colonies stained with crystal violet formed by the indicated clones after 14 days. (**C**) A transwell migration assay was performed to detect the migratory capacity of Shoc2 siRNA-treated cells. Representative image of transwell assay of SW480 cells transfected with control siRNA or Shoc2 siRNA.

To determine the effects of Shoc2 down-regulation on cell proliferation, CCK-8 assays were performed on the Shoc2 knockdown (Shoc2 siRNA) and the control siRNA. Shoc2 knockdown in SW480 cell line resulted in significant reduction in cell proliferation (Figure 5A). In addition, colony-forming ability of Shoc2 siRNA and control siRNA was assessed. Shoc2 knockdown clones demonstrated considerable reduction in colony-forming efficiency compared with the control clones (Figure 5B). To investigate whether Shoc2 knockdown inhibits the migration of SW480 cells, we performed a transwell migration assay. Cell mobility was significantly decreased in Shoc2 siRNA-transfected cells compared with the control siRNA-transfected cells (Figure 5C). Our results therefore suggest that Shoc2 knockdown leads to substantial reduction in cell proliferation and mobility.

### Effect of Celastrol in other CRC cell lines cell viability and acute mice toxicity study

**Figure 6:**
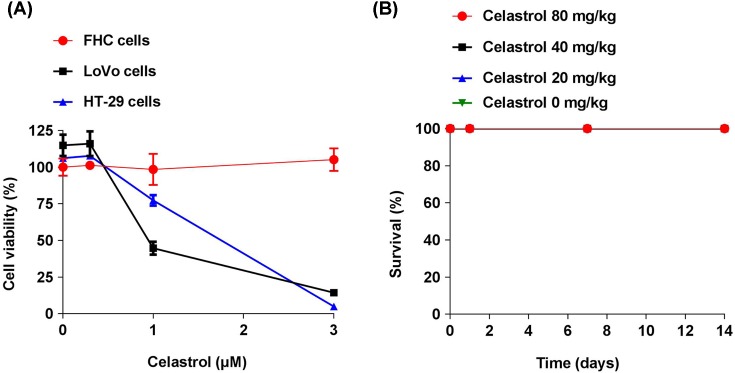
Effect of Celastrol in cell viability and acute toxicity studies (**A**) Quantitative data of cell viability under different concentrations of Celastrol treatments for 24 h, *n*=6. (**B**) Male mice (n=10 per group) were treated with Celastrol (80, 40, 20, or 0) mg/kg by gavage and lethality was evaluated every 12 h during 14 days. Results are expressed as percentage of survival.

To determine the effects of Celastrol on additional CRC cell lines and normal cells viability, a fetal colon cell line FHC cells and two other CRC cell lines LoVo cells and HT-29 cells were used in the present study. Celastrol (1 and 3 μM) significantly decreased LoVo and HT-29 cells viability, but not FHC cells (Figure 6A). Acute mice toxicity study revealed that the LD_0_ of Celastrol by gavage is more than 80 mg/kg (Figure 6B).

## Discussion

In the present study, we demonstrate that Celastrol exerted anti-CRC functions in a CRC cell line *in vitro* and a xenograft tumor model *in vivo*. Regarding the study of mechanisms, we combined biotinylated Celastrol and the human protein microarray together to identify Celastrol-binding proteins. We confirmed 69 Celastrol-binding proteins through this method. Of these proteins, Shoc2 was significantly enriched. Celastrol inhibited the phosphorylation of the Shoc2 downstream protein ERK. In addition, down-regulation of Shoc2 expression inhibited ERK phosphorylation and significantly inhibited proliferation and migration of CRC cells. In summary, our study is the first to confirm that Celastrol exerts anti-tumor functions in human CRC at least partially by inhibiting ERK1/2 phosphorylation via its binding to Shoc2.

Previous studies reported that Celastrol exhibited apoptosis-inducing function in cancer cells in both cell and animal models [21–24]. Lu et al. [25] confirmed that Celastrol promoted apoptosis in another CRC cell line, HT-29, through β-catenin. Yadav et al. [26] elucidated that Celastrol inhibited CRC cell invasion and metastasis through down-regulation of C-X-C chemokine receptor type 4 expression. Although the mechanism of action for Celastrol in CRC has been preliminarily confirmed [27,28], one of the most effective methods for elucidating the mechanism of action of a drug is to identify its binding proteins [29,30]. The high-throughput proteomics method provides a powerful tool to systematically confirm the targets of anti-cancer drugs. We used the proteome microarray containing 16368 proteins and Bio-Celastrol to confirm 69 candidate targets of Celastrol. In addition, we validated that Shoc2 was a direct target of Celastrol. These results confirmed the feasibility and specificity of this strategy. Therefore, proteomics combined with labeling technology can help to reveal the direct target of most compounds.

Shoc2 is a conserved protein containing leucine-rich repeats. Shoc2 knockout in mice results in embryonic lethality [31]. SHOC2 mutations are identified in Noonan syndrome patients [32]. These results indicate the important role of Shoc2 in embryonic development. Shoc2 plays a critical role in ERK-MAPK pathway activation [33]. The ERK-MAPK pathway is up-regulated in the majority of human cancers [34,35]. Shoc2 activates ERK1/2 to promote tumor development through regulation of contact inhibition and cell polarization [34]. Kaduwal et al. [36] confirmed that Shoc2 regulates motility, invasion, and metastasis of cells through activation of the ERK and PI3K pathways. These results indicated that Shoc2 might play an important role in tumorigenesis and that inhibition of Shoc2 functions may be a promising novel anti-cancer strategy. We demonstrated that inhibition of Shoc2 by Celastrol reduced ERK1/2 phosphorylation and that down-regulation of Shoc2 significantly inhibited tumor growth. These results indicated that Shoc2 might be a functional target of the biological function of Celastrol.

In summary, we comprehensively investigated the proteins directly binding to Celastrol. The confirmed Celastrol-binding proteins in the present study provided valuable resources. Our results elucidated the significant anti-CRC function of Celastrol, which was mediated by its inhibition of the Shoc2-ERK1/2 signaling pathway. This target might represent the general mechanism underlying the inhibitory function of Celastrol on cancer cells.

## Supporting information

**Table:** 

## Abbreviations

CRC: colorectal cancer
CXCR4: C-X-C chemokine receptor type 4
ERK: extracellular-regulated protein kinases
MAPK: mitogen-activated protein kinase
OD: optical density
