# Correction: Systematic identification of celastrol-binding proteins reveals that Shoc2 is inhibited by celastrol

**DOI:** 10.1042/BSR-20181233_COR

**Published:** 2021-04-27

**Keywords:** Celastrol, Colorectal cancer, ERK, Human proteome microarray, Shoc2

The authors of the original article “Systematic identification of Celastrol-binding proteins reveals that Shoc2 is inhibited by Celastrol” (*Biosci Rep* (2018) 38(6), **DOI**: 10.1042/BSR20181233) would like to correct Figure 1 of their article. During figure preparation for the submission of their manuscript, their NC image file had inadvertently been used twice, and the DMSO image had been labelled as the 0.1 image in error. The correct version of Figure 1C is present in this Correction. The authors apologise for the errors made and for any inconvenience caused. They confirm that these figure corrections do not impact the data interpretation and conclusions of their paper.

**Figure 1:**
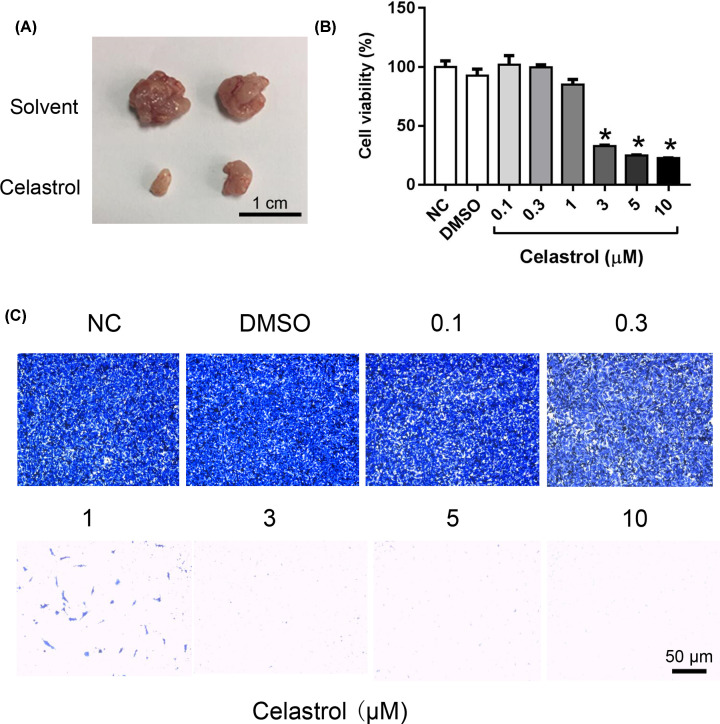
Celastrol attenuates the tumorigenicity of colon cancer cell *in vivo* and *in vitro* (**A**) Representative images of tumor tissues harvested from different groups. Mice were given Celastrol by gavage at a dose of 3 mg/kg daily for 15 days, *n* = 4. The solvent formulation: 10% DMSO, 70% cremophor/alcohol (3:1), and 20% PBS. (**B**) Quantitative data of cell viability under different concentrations of Celastrol treatments for 24 h, *n* = 6, *P<0.001 vs. DMSO group. (**C**) SW480 cells were transfected with different concentrations of Celastrol. Cell migration was assessed after 24 h incubation by transwell assay, *n* = 6.

