# Supplementary figures and images for: Systematic identification of Celastrol-binding proteins reveals that Shoc2 is inhibited by Celastrol
Source: Biosci Rep. 2018 Nov 21;38(6):BSR20181233. doi: 10.1042/BSR20181233 (PMC6246769; doi:10.1042/BSR20181233)

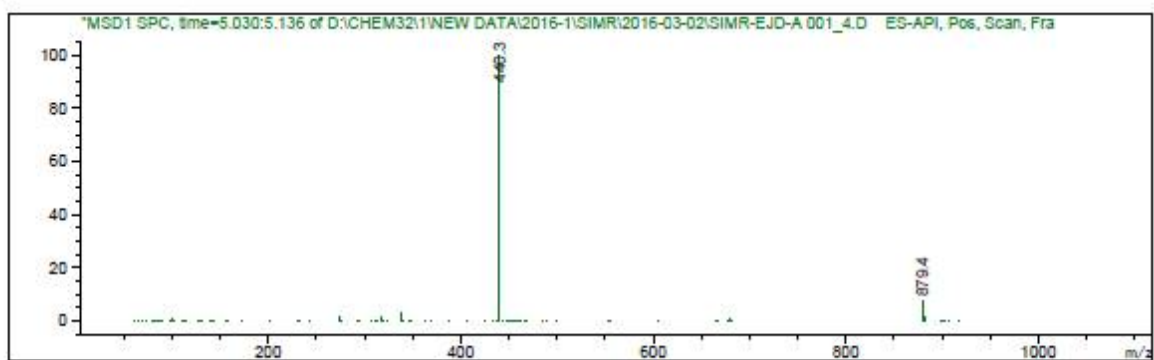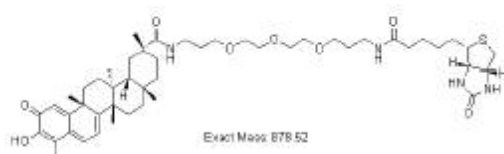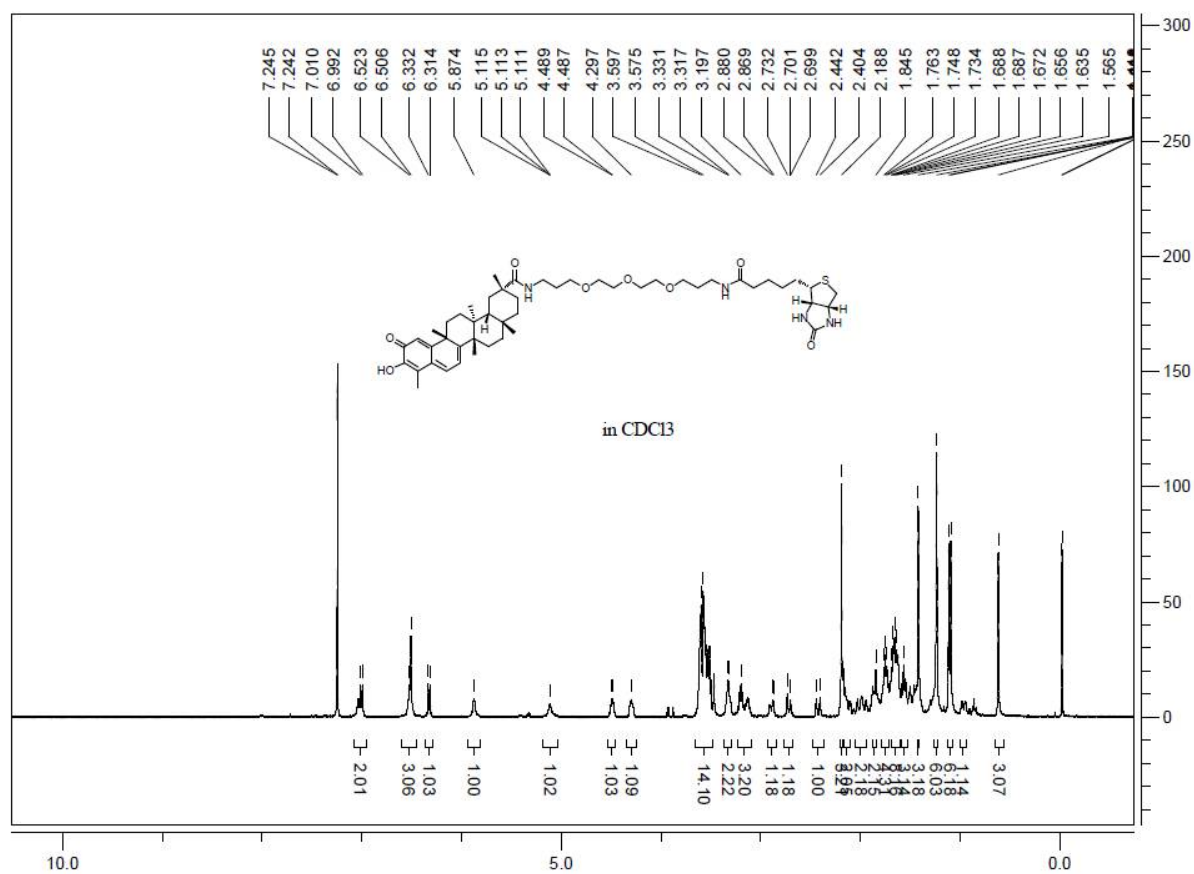

Supplement: Supplementary file 1 [file bsr20181233_Supp1.pdf]
